# Microglial Ramification, Surveillance, and Interleukin-1β Release Are Regulated by the Two-Pore Domain K^+^ Channel THIK-1

**DOI:** 10.1016/j.neuron.2017.12.002

**Published:** 2018-01-17

**Authors:** Christian Madry, Vasiliki Kyrargyri, I. Lorena Arancibia-Cárcamo, Renaud Jolivet, Shinichi Kohsaka, Robert M. Bryan, David Attwell

**Affiliations:** 1 Department of Neuroscience, Physiology, and Pharmacology, University College London, Gower Street, London WC1E 6BT, UK; 2 Institute of Neurophysiology, Charité – Universitätsmedizin, 10117 Berlin, Germany; 3 CERN and Département de physique nucléaire et corpusculaire, University of Geneva, 1211 Geneva 4, Switzerland; 4 National Institute of Neuroscience, 4-1-1 Ogawa-Higashi, Kodaira, Tokyo 187-8502, Japan; 5 Department of Anesthesiology, Baylor College of Medicine, 434D Baylor College of Medicine, One Baylor Plaza, Houston, TX 77030, USA

**Keywords:** microglia, potassium channel, ATP, surveillance, inflammasome, interleukin-1β, ramification, THIK-1

## Abstract

Microglia exhibit two modes of motility: they constantly extend and retract their processes to survey the brain, but they also send out targeted processes to envelop sites of tissue damage. We now show that these motility modes differ mechanistically. We identify the two-pore domain channel THIK-1 as the main K^+^ channel expressed in microglia *in situ*. THIK-1 is tonically active, and its activity is potentiated by P2Y_12_ receptors. Inhibiting THIK-1 function pharmacologically or by gene knockout depolarizes microglia, which decreases microglial ramification and thus reduces surveillance, whereas blocking P2Y_12_ receptors does not affect membrane potential, ramification, or surveillance. In contrast, process outgrowth to damaged tissue requires P2Y_12_ receptor activation but is unaffected by blocking THIK-1. Block of THIK-1 function also inhibits release of the pro-inflammatory cytokine interleukin-1β from activated microglia, consistent with K^+^ loss being needed for inflammasome assembly. Thus, microglial immune surveillance and cytokine release require THIK-1 channel activity.

## Highlights

• The two-pore domain channel THIK-1 is the main K^+^ channel in “resting” microglia
• Tonic activity of THIK-1 maintains the microglial resting potential
• Blocking THIK-1 reduces microglial ramification, surveillance, and IL-1β release
• Surveillance depends on THIK-1, not P2Y_12_; chemotaxis depends on P2Y_12_, not THIK-1

## Teaser

Microglia survey the brain for invading micro-organisms, remove dying neurons, and prune synapses during development. We show that maintenance of the microglial resting potential by THIK-1 K^+^ channels is essential for maintaining microglial ramification, surveillance, and interleukin-1β release.

## Introduction

Microglia continuously extend and retract their fine processes in the healthy brain (Nimmerjahn et al., 2005, Davalos et al., 2005). This process movement, henceforth termed “surveillance” of the brain, is assumed to play a key role in monitoring the ingress of bacteria, fungi, and viruses (Hanisch and Kettenmann, 2007); detecting the release of ATP from damaged cells (Davalos et al., 2005); and sensing entry of fibrinogen into the brain’s extracellular space from damaged blood vessels (Davalos et al., 2012). However, microglial surveillance also plays an important role in monitoring synaptic function and determining the “wiring” of the brain (Wake et al., 2009, Tremblay et al., 2010, Schafer et al., 2012). During postnatal development, synapses that are to be pruned become tagged with complement molecules and are thus removed by microglia (in the dorsal lateral geniculate nucleus; Schafer et al., 2012, Stevens et al., 2007). Disruption of this system leads to altered wiring of the CNS, generating an excess of excitatory synapses that promotes epilepsy (Chu et al., 2010) and neuropsychiatric disorders (Zhan et al., 2014), while during ischemia the interaction of microglia with synapses is markedly prolonged and may lead to a loss of synapses (Wake et al., 2009). Furthermore, in the healthy brain, microglia preferentially contact neurons with high levels of activity and decrease their firing rate (Li et al., 2012). All of these functions presumably depend on microglia sensing their environment by repeatedly extending and retracting their processes, but the factors regulating microglial surveillance are unknown.

Movement of microglial processes to sites of tissue damage is known to depend on the activation of microglial P2Y_12_ receptors by ATP (or ADP derived by its hydrolysis) released from the damage site (Haynes et al., 2006) and may involve cytoskeletal changes driven by P2Y_12_ activating integrin-β1 (Ohsawa et al., 2010). In contrast, the constant surveillance of the brain by microglia is unaffected by knockout (KO) of P2Y_12_ (Haynes et al., 2006, Sipe et al., 2016), implying that it is controlled by a different mechanism. By patch clamping microglia in brain slices and imaging their movements in brain slices and *in vivo*, we now demonstrate that the two motility modes of microglia—directed process movement to a damage site and ceaseless surveillance of the brain—are differentially controlled by P2Y_12_ activation and by membrane potential. By characterizing the membrane current activated by P2Y_12_ receptors, we show for the first time that the microglial resting potential is maintained by a two-pore domain K^+^ channel that we identify as THIK-1 (TWIK-related Halothane-Inhibited K^+^ channel), the product of the *Kcnk13* gene (Rajan et al., 2001), and demonstrate that this channel is tonically active even without ATP or ADP present to activate P2Y_12_ receptors. We show that the tonic activity of THIK-1 is crucial for maintaining normal immune surveillance by microglia, by maintaining their ramified morphology. In addition, we show that the activity of THIK-1 is essential for microglial generation of the inflammatory mediator interleukin-1β.

## Results

### Membrane Currents and Purinergic Signaling Associated with Brain Damage

**Figure 1:**
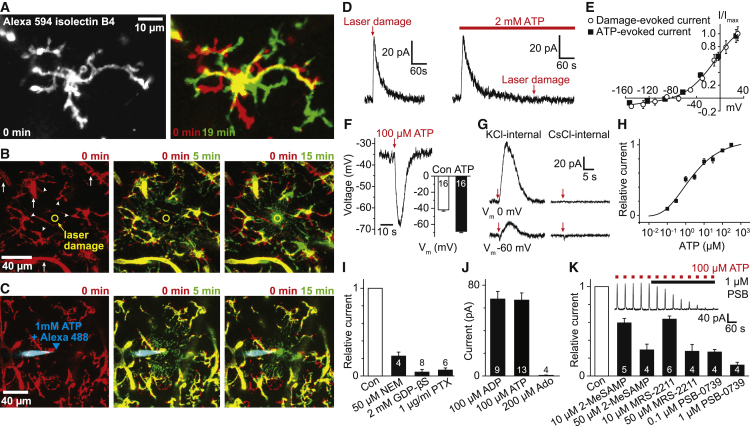
Microglial Surveillance, Directed Motility, and Damage-Evoked Membrane Current in Hippocampal Slices (A) Left: Microglial cell labeled with Alexa 594-isolectin B_4_. Right: Superimposed images of the same cell (green and red) at an interval of 19 min, showing process movement during surveillance (red, retracted; green, extended processes; same convention in subsequent time-lapse panels). (B) Microglial processes (arrowheads) converging on a laser-damaged region (10 μm circle, time after damage indicated). Blood vessels (arrows) are also labeled by isolectin B_4_. (C) Microglial processes converging on tip (arrowhead) of a pipette filled with 1 mM ATP (and Alexa 488, recolored blue). (D) Patch-clamped microglial membrane current at 0 mV evoked by laser damage (left) is mimicked and occluded by 2 mM ATP (right). (E) Voltage dependence of laser damage- and ATP-induced currents in 5 and 9 microglia, respectively. (F) ATP (puffed at arrow) evokes hyperpolarization of microglia (inset: mean values differ significantly, p = 1.2 × 10^−12^). (G) 100 μM ATP-evoked currents at 0 and −60 mV with K^+^ and Cs^+^ in pipette. (H) Dependence of ATP-evoked current at 0 mV on puffed [ATP]. (I) Effect on ATP-evoked current of bath applied N-ethyl-maleimide (NEM, 50 μM, p = 6.7 × 10^−5^, paired t test), GDPβS in the patch pipette (2 mM replacing 0.5 mM GTP, p = 3.4 × 10^−10^, unpaired t test), and pertussis toxin (PTX, 1 μg/ml, control and pertussis-exposed slices were incubated for 24 hr at 37°C, p = 4.6 × 10^−6^, unpaired t test). (J) Mean currents evoked at 0 mV by puffed ATP and ADP (both 100 μM), and superfused 200 μM adenosine. (K) ATP/ADP-evoked current at 0 mV is blocked by 0.1 or 1 μM PSB-0739 (PSB, p = 6.7 × 10^−4^ and 6.2 × 10^−4^), 10 or 50 μM MRS-2211 (p = 6.1 × 10^−4^ and 2.6 × 10^−3^), and 10 or 50 μM 2-MeS-AMP (p = 4.0 × 10^−3^ and 3.2 × 10^−3^); p values from paired t tests. Inset shows block of ATP-evoked current by PSB-0739. p values, here and in other figures, were corrected for multiple comparisons within each panel. All data are from P12 rat. Data are represented as mean ± SEM. See also Figure S1.

To investigate the mechanisms of microglial surveillance, we studied microglia labeled with fluorescently tagged isolectin B_4_ in rats or mice, or, where stated, genetically labeled with eGFP under control of the Iba1 promoter in mice (see STAR Methods). Since microglia in culture can express proteins different from those *in situ* (Boucsein et al., 2003, Butovsky et al., 2014, Bohlen et al., 2017, Gosselin et al., 2017), experiments were on microglia *in situ* in acute hippocampal brain slices (to allow pharmacological analysis of mechanisms) or *in vivo* in cortex (to confirm the role of THIK-1 *in vivo*; see STAR Methods). As previously reported (Nimmerjahn et al., 2005, Davalos et al., 2005), two-photon imaging revealed that microglia display continual process extension and retraction in all directions under physiological conditions, allowing gradual surveillance of the brain (Figures 1A and S1A; Movie S1), and promptly extend processes toward a site of laser-induced cell damage (Figure 1B; Movie S2) or toward an ATP source mimicking the ATP released from dying cells (Figure 1C; Movie S2). Patch clamping was used to investigate the membrane currents associated with these different kinds of motility and their role in regulating surveillance by microglia. Experiments were carried out less than 4 hr after brain slicing, on microglia located 50–100 μm deep in the slice, to avoid microglial activation (Hanisch and Kettenmann, 2007, Kurpius et al., 2006).

Microglia *in situ* in rat brain slices had a mean resting potential of −40.6 ± 0.6 mV (n = 151), which is more depolarized than neurons or other glia, and a high input resistance of 2.1 ± 0.1 GΩ, implying that small membrane current changes will have a large effect on the membrane potential. They showed time-independent currents in response to brief voltage steps away from the resting potential (Figures S1B–S1C), indicating a lack of voltage-gated channel activity in microglia *in situ* in the healthy brain. Laser-induced damage to cells in the slice evoked a membrane current in microglia that showed outward rectification and a reversal potential near the Nernst potential for K^+^ (E_K_) and was mimicked and occluded by superfusion of the slice with 2 mM ATP (Figures 1D and 1E), suggesting that the damage-induced K^+^ current is activated by ATP (or a derivative) released from damaged cells.

Locally puffing 100 μM ATP to mimic its release from damaged cells (see STAR Methods) hyperpolarized microglia by ∼30 mV (Figure 1F). In voltage-clamp mode, ATP evoked an outwardly rectifying membrane current reversing near E_K_, which resembles that induced by laser damage (Figure 1E, current density 3.84 ± 0.14 pA/pF at −4 mV, n = 103). This current was abolished when K^+^ in the pipette was replaced with Cs^+^ (Figure 1G) and desensitized very slowly in response to prolonged ATP application (Figure 1D, τ = 54.1 ± 7.8 s at 36°C, n = 6). At negative membrane potentials, this K^+^ current was sometimes preceded by a small inward current, which reversed around 0 mV (Figure 1G). These currents have previously been suggested to reflect G protein-coupled P2Y and ionotropic P2X receptor activation, respectively (Boucsein et al., 2003, Wu et al., 2007). The K^+^ current has a large effect on the membrane potential, but its role in regulating microglial motility and cytokine release is unknown.

The ATP-evoked K^+^ current was activated with an apparent EC_50_ of ∼2 μM (for the [ATP] in the puffing pipette; Figure 1H), and was inhibited by N-ethyl-maleimide or pertussis toxin or by including GDPβS in the recording pipette (Figure 1I), suggesting the involvement of a G_i_ protein-coupled receptor. Candidate microglial receptors for ATP and its derivatives from transcriptome data (Zhang et al., 2014) include the nucleoside phosphate receptors P2Y_12_, P2Y_13_, P2Y_6_, and P2Y_2_, as well as receptors for adenosine. The K^+^ current was also evoked by the ATP breakdown product ADP, which is a P2Y_12_/P2Y_13_ agonist, but not by adenosine (Figure 1J). It was inhibited (Figure 1K) by 0.1–1 μM PSB-0739 (which blocks P2Y_12_ but not P2Y_13_ or P2Y_2_; Hoffmann et al., 2009) and by 10–50 μM MRS-2211 and 10–50 μM 2-MeS-AMP (which block P2Y_12_ and P2Y_13_). It was not blocked by the P2Y_6_ antagonist MRS-2578 (10 μM, increased by 2.0% ± 4.5% in 5 cells, p = 0.78) or the P2Y_2_ antagonist AR-C 118925XX (50 μM, increased by 8.8% ± 7.3% in 4 cells, p = 0.3). Thus, the K^+^ current is evoked by ATP or ADP acting on microglial P2Y_12_ receptors (Swiatkowski et al., 2016), which also mediate microglial process extension toward a localized ATP source or tissue damage (Haynes et al., 2006).

### P2Y_12_ Receptors Gate the Two-Pore Domain K^+^ Channel THIK-1

**Figure 2:**
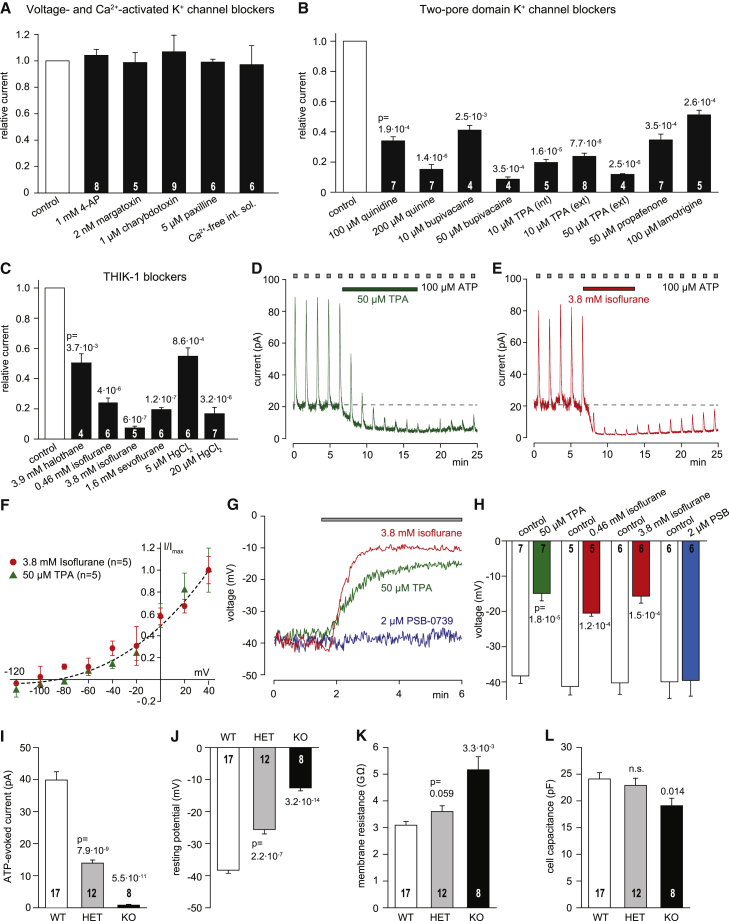
ATP and ADP Gate an Anesthetic-Sensitive Tonically Active Two-Pore Domain K^+^ Channel (A–C) Effect on the 100 μM ATP-evoked current at 0 mV, normalized to control data in the same cell, of the following agents. (A) Blockers of voltage- and calcium-gated K^+^ channels. (B) Blockers of two-pore domain channels (quinidine and quinine also block voltage-gated K^+^ channels, and bupivacaine, propafenone and lamotrigine also block voltage-gated Na^+^ channels, but all block two-pore K^+^ channels). (C) Effects of gaseous anesthetics and Hg^2+^. (D and E) Response to repeatedly puffed ATP (100 μM) during superfusion of (D) tetrapentylammonium or (E) isoflurane shows a suppression of baseline current and of the response to ATP. (F) Voltage dependence of the baseline current suppressed by tetrapentylammonium and isoflurane. (G) Effect of tetrapentylammonium, isoflurane, and PSB-0739 on the microglial resting potential. (H) Mean resting potential in the agents in (G), compared with the resting potential before the drug was applied (control). The lower concentration of isoflurane (0.46 mM) is a level reached in anesthesia. (A–H) are from P12 rat. (I–L) Values of (I) ATP-evoked current, (J) membrane potential, (K) membrane resistance, and (L) cell capacitance for microglia in P15-22 mice that are WT, heterozygote, or KO for THIK-1. Numbers of cells are on bars. Data are from hippocampal slices, and are represented as mean ± SEM. See also Figures S1, S4, and S5.

To examine the functional role of these microglial K^+^ channels, we first defined their pharmacology. The outward-rectifying current-voltage relation of the K^+^ current (Figure 1E) excludes it being mediated by a member of the inward-rectifying K^+^ channel family, but is consistent with activation of delayed rectifier or Ca^2+^-activated K^+^ channels or of two-pore domain K^+^ channels. Blocking voltage-activated (including Kv1.3) and Ca^2+^-activated channels (with 4-aminopyridine [4-AP] 1 mM, margatoxin 2 nM, charybdotoxin 1 μM, paxilline 5 μM, or omission of Ca^2+^ from the pipette solution) had no effect on the ATP-evoked current (Figure 2A). In contrast, six agents that block two-pore domain K^+^ channels (Lotshaw, 2007, Piechotta et al., 2011)—quinidine (100 μM), quinine (200 μM), bupivacaine (10–50 μM), tetrapentylammonium (TPA, 10–50 μM), propafenone (50 μM), and lamotrigine (100 μM)—all greatly reduced the current (Figure 2B).

RNA profiling (Butovsky et al., 2014, Zhang et al., 2014, Hickman et al., 2013) of microglia indicates high expression of the two-pore domain family members TWIK-2 (Tandem of p domains in a Weak Inward rectifying K^+^ channel 2), THIK-1, and THIK-2. Of these, TWIK-2 and THIK-2 alone are unlikely to mediate the current in Figures 1D and 1E because TWIK-2 generates a weakly inward-rectifying and rapidly desensitizing (rather than outwardly rectifying and slowly desensitizing) current and is not inhibited by bupivacaine (Lotshaw, 2007), while THIK-2 may largely reside in the endoplasmic reticulum (Renigunta et al., 2014). Unlike most other two-pore domain channels, the remaining candidate THIK-1 is blocked by the gaseous anesthetics halothane and isoflurane rather than being activated by these agents (Rajan et al., 2001, Lotshaw, 2007). We found that the ATP-evoked current was inhibited by halothane (3.9 mM) and also by the structurally related gaseous anesthetics sevoflurane (1.6 mM) and isoflurane (3.8 mM and 460 μM, a level reached during clinical anesthesia; Franks and Lieb, 1996) (Figure 2C). Similarly, the current was reduced by mercury (Hg^2+^, 5–20 μM; Figure 2C), which inhibits THIK-1 but potentiates or has no effect on most other two-pore domain K^+^ channels (Lotshaw, 2007). Thus, P2Y_12_ receptors gate K^+^ channels containing THIK-1 subunits, which are highly expressed in microglia (and to a lesser extent in oligodendrocytes, but not in neurons or astrocytes; Zhang et al., 2014).

### THIK-1 Is Tonically Active

Although THIK-1 can be activated by ATP or ADP, we found that this channel is tonically active even without added extracellular ATP. Applying tetrapentylammonium (Figure 2D) or isoflurane (Figure 2E; even at low concentrations used for anesthesia; see below) suppressed a membrane current that had the same I-V relation as the THIK-1-mediated current (Figure 2F) and thus depolarized microglia by ∼25 mV (Figures 2G and 2H). In contrast, blocking P2Y_12_ receptors with PSB-0739 did not affect the baseline membrane current or membrane potential of microglia (Figure 1K, inset; Figures 2G and 2H), implying that P2Y_12_ is not tonically active and hence that THIK-1 itself is inherently tonically active (or its activity is maintained by the activity of a receptor other than P2Y_12_).

Confirming our pharmacological analysis, KO of THIK-1 completely abolished the ATP-evoked K^+^ current (Figure 2I), indicating that this current is mediated by channels containing THIK-1 subunits (although we cannot rule out a heterodimer [Lotshaw, 2007] of THIK-1 with THIK-2 or TWIK-2) and implying that THIK-1 is essential for the K^+^ efflux evoked by tissue damage. THIK-1 KO also depolarized the resting potential (Figure 2J) to −12 mV (similar to the resting potential seen in the presence of THIK-1 blockers; Figure 2H) and increased the cell membrane resistance (Figure 2K), confirming that THIK-1 is tonically active in the absence of added ATP or ADP. KO of THIK-1 also decreased microglial capacitance (Figure 2L), which we will show reflects a change in cell morphology when THIK-1 is blocked.

Consistent with tonically active THIK-1 contributing most of the K^+^ conductance of non-activated microglia, superfusing 22.5 mM [K^+^]_o_ solution onto hippocampal slices from wild-type (WT) mice evoked a large microglial depolarization (ΔV_m_ = +13.9 ± 1.4 mV, n = 6), unlike for microglia in THIK-1 KO slices (ΔV_m_ = −4.2 ± 0.8 mV, n = 6, significantly less, p = 5.6 × 10^−7^; the small hyperpolarization in KO cells may reflect high [K^+^]_o_ activating the Na/K pump to generate an outward membrane current).

Thus, tonically active THIK-1 channels, but not P2Y_12_ receptors, maintain much of the resting potential of microglia.

### THIK-1 Regulates Microglial Ramification and Surveillance but Not Directed Motility

**Figure 3:**
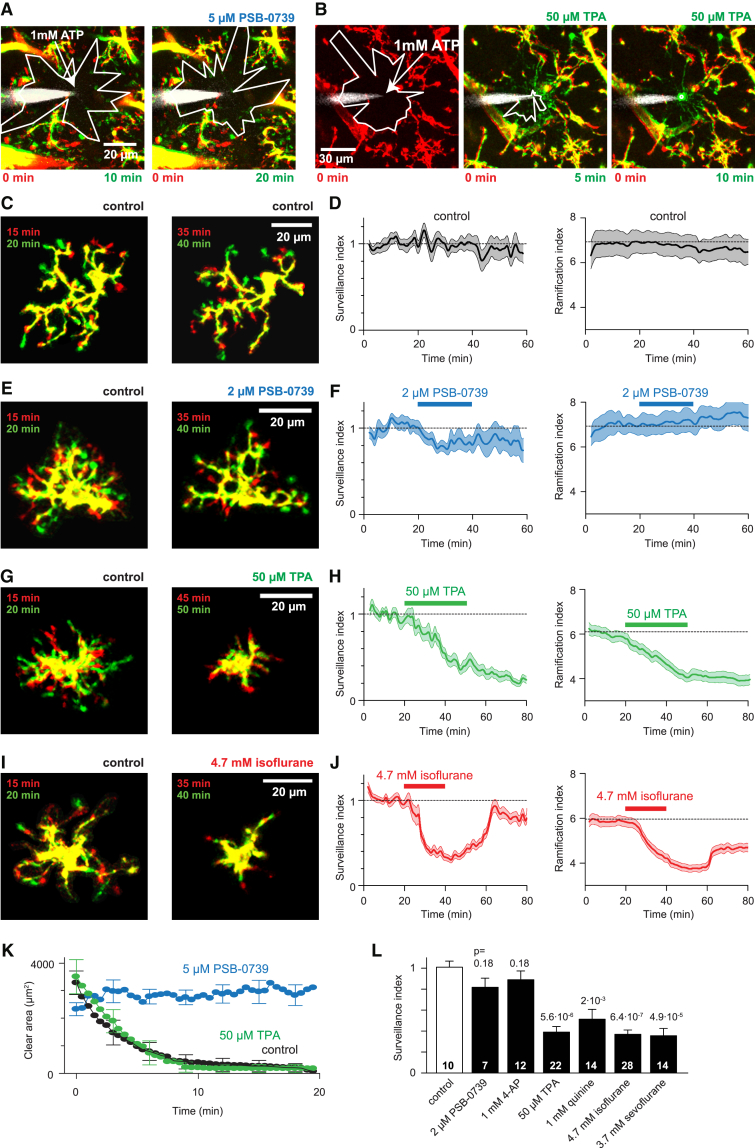
Effect on Directed Motility and Surveillance of P2Y_12_ and THIK-1 Block in Hippocampal Slices (A and B) Effect of (A) PSB-0739 (PSB) to block P2Y_12_ receptors (contrast with Figure 1C) and (B) tetrapentylammonium (TPA) to block THIK-1 channels, on directed motility (quantified in K) toward a pipette (arrow) filled with 1 mM ATP (and Alexa 488, white, for visualization). Images taken at times shown after placing the pipette; colors as in Figure 1A. (C and D) Long-term stability of surveillance revealed by (C) images taken 5 min apart in control conditions, showing many process extensions (green) and retractions (red), and (D) time course of surveillance and ramification indices (see STAR Methods). (E and F) As for (C) and (D) but with application of PSB-0739 to block P2Y_12_, showing no significant effect (see L) on surveillance and ramification. (G–J) As for (E) and (F) but applying TPA (G and H) or isoflurane (I and J) to block THIK-1, showing reduced ramification and fewer process extensions and retractions with THIK-1 blocked. (K) Time course of directed motility quantified as reduction of the “clear area” not occupied by microglia around a laser-damaged spot (white polygon on A and B; see STAR Methods and Movie S3) in control conditions (n = 10) and with PSB (n = 6) or TPA (n = 7) present. (L) Mean effects of drugs on surveillance (averaged over last 5 min in each drug). Number of microglia shown on bars; p values compared with control data (white bar, averaged 35–40 min in D) were from Mann-Whitney tests. A higher [PSB] is used in (A) than in (E), because PSB blocks P2Y_12_ receptors competitively (Hoffmann et al., 2009) and a high [ATP] is used in (A). All data are from P12 rat. Data are represented as mean ± SEM. See also Figures S1 and S4.

To assess the physiological significance of the tonic activity of THIK-1 and the hyperpolarization that it produces, we imaged microglial motility in brain slices as in Figure 1. Attraction of processes to an ATP source, surveillance, and ramification of the microglial processes were quantified as described in the STAR Methods. Briefly, the surveillance index is a measure of the number of image pixels surveyed per unit time and depends both on the number of cell processes and on their speed and range of movement, while the ramification index is a measure of the ratio of the cell’s perimeter to its area (normalized to that of a circle of the same area) and depends on the cell’s shape, but not on its overall size. Blocking P2Y_12_ receptors with PSB-0739 prevented the directed motility (chemotaxis) evoked by an ATP source (Figure 3A, quantified in Figure 3K; Movie S3), but did not significantly affect microglial morphology or surveillance of the brain by microglia (Figures 3E, 3F, and 3L; Movie S4) as found previously with P2Y_12_ knockout or knockdown (Haynes et al., 2006, Sieger et al., 2012, Sipe et al., 2016). In contrast, blocking THIK-1 with tetrapentylammonium did not affect directed motility (Figure 3B, quantified in Figure 3K; Movie S3) but evoked retraction of microglial processes and inhibited surveillance by ∼60% (Figures 3G, 3H, and 3L; Movie S4; changes in neuronal spiking were prevented by having 0.5 μM TTX present throughout, which does not affect microglial surveillance [Nimmerjahn et al., 2005] or directed motility [Hines et al., 2009]). A similar effect on microglial surveillance was evoked by the other THIK-1 blockers quinine, isoflurane, and sevoflurane (Figures 3I, 3J, and 3L), whereas imaging for the same period in control solution (Figures 3C and 3D) or applying the voltage-gated K^+^ channel blocker 4-AP (Figure 3L) had no effect on surveillance. Thus, THIK-1 activity is essential for the maintenance of microglial ramification and surveillance.

Our observation that blocking THIK-1 with 50 μM tetrapentylammonium does not affect directed motility (Figure 3B, quantified in Figure 3K) contradicts an unquantified study claiming that blocking microglial ATP-gated K^+^ channels with 1 mM quinine prevents directed motility (Wu et al., 2007). The latter effect of quinine may reflect the intracellular alkalinization that it produces or its effects on gap junctional hemichannels or the cytoskeleton (Dixon et al., 1996, Yoshida and Inouye, 2001).

**Figure 4:**
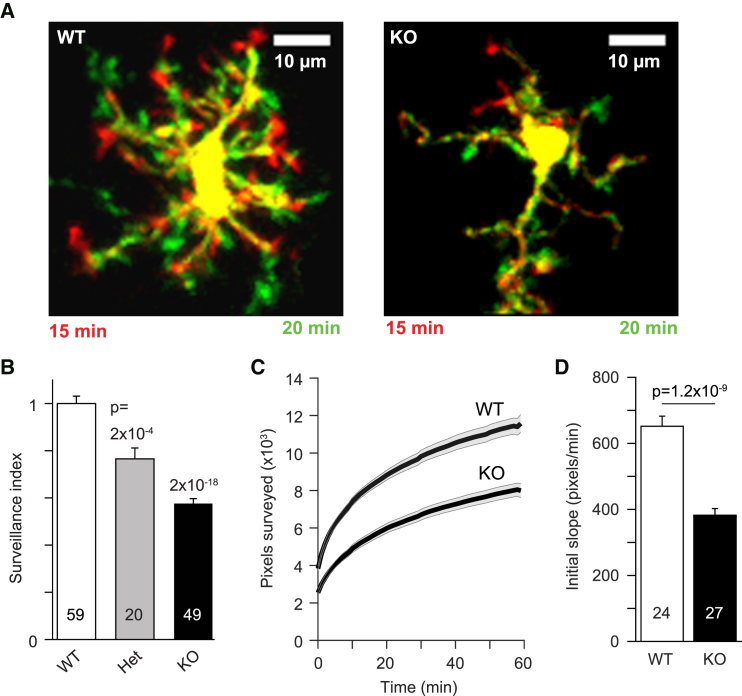
Effect of THIK-1 Knockout on Microglial Surveillance, Morphology, and Density in Hippocampal Slices (A) Specimen images taken 5 min apart of WT (P27) and THIK-1 KO Iba1-GFP (P21) microglia, showing process extensions and retractions (colors as in Figure 1A) and the less ramified shape of microglia in the KO. (B) Quantification of surveillance for microglia from P20–P27 WT, heterozygote (Het), and KO mice, showing increasing inhibition of surveillance from Het to KO. (C) Time course of increase of the number of surveyed pixels in maximum-intensity projections of images of microglia (as in Movie S5, numbers of cells on bars in D) in WT and KO microglia in Iba1-GFP mice aged P20–P27. Initial value is the area of the cell in the first image frame. (D) Initial slope of graphs in (C) (measured over the first 2 min, when assessment of surveillance is least confounded by pixel overlap in the maximum-intensity projection). Data are represented as mean ± SEM. See also Figures S2 and S5.

**Figure 5:**
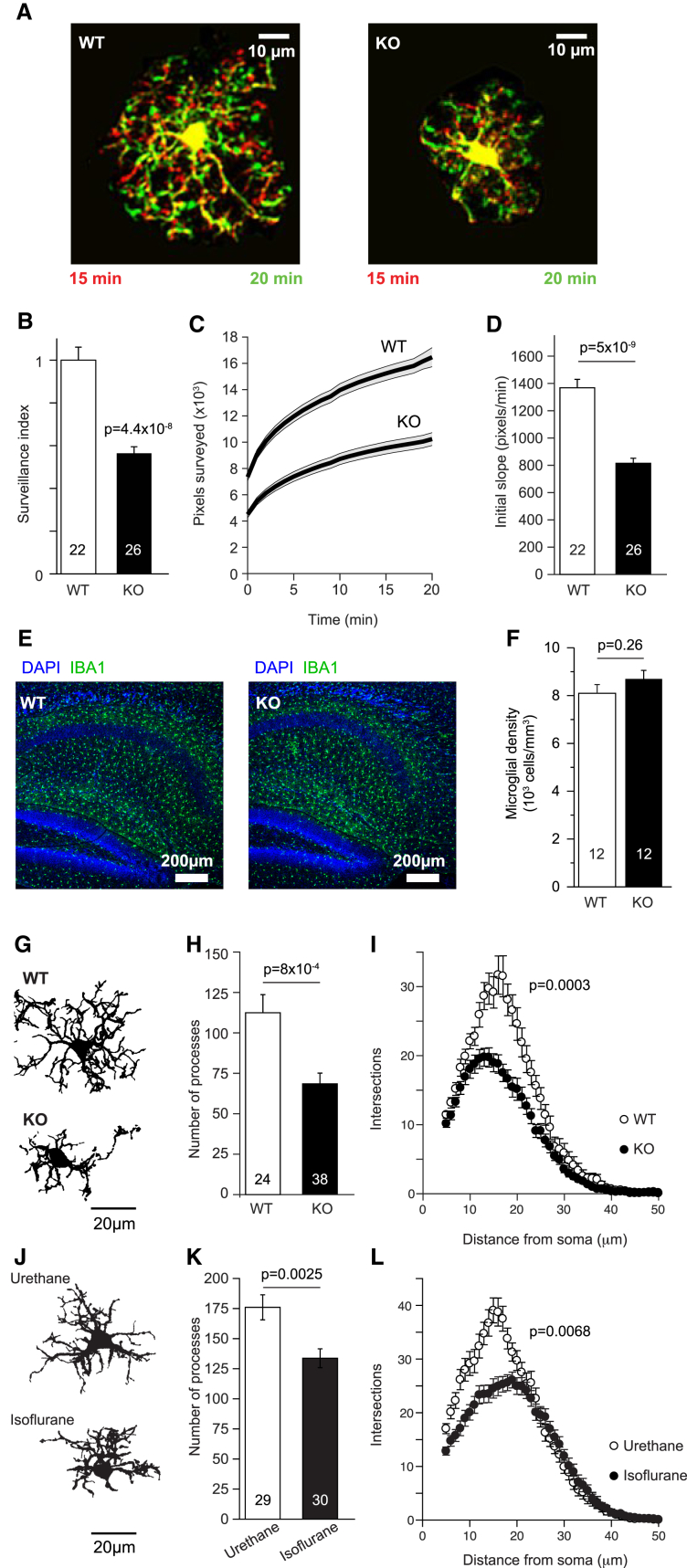
Effect of THIK-1 Knockout on Microglial Surveillance, Morphology, and Density, *In Vivo* (A) Specimen images taken 5 min apart *in vivo* of WT (P23) and THIK-1 KO Iba1-GFP (P22) microglia, showing process extensions and retractions (colors as in Figure 1A) and the less ramified shape of microglia in the KO. (B) Quantification of surveillance for microglia from P21–P27 WT and KO mice, showing less surveillance in the KO. (C) Time course of increase of the number of surveyed pixels in maximum-intensity projections of images of microglia (as in Movie S5, numbers of cells on bars in D) in WT and KO Iba1-GFP mice aged P21–P27. Initial value is the area of the cell in the first image frame. (D) Initial slope of graphs in (C) (measured over the first 2 min, when assessment of surveillance is least confounded by pixel overlap in the maximum-intensity projection). (E) Specimen images of perfusion-fixed WT and THIK-1 KO hippocampal slices labeled for Iba1 show that microglial density and tiling appear unchanged in the KO. (F) Microglial density in the strata radiatum and lacunosum-moleculare of areas CA1-CA3 of 12 WT and 12 KO hippocampal slices (from 3 WT and 3 KO animals at P20–P27). (G–I) Ramification analysis of P17–P21 microglia from perfusion-fixed WT and THIK-1 KO mice showing (G) representative 3D-reconstructed WT and KO microglia, and (H and I) Sholl analysis-derived number of processes (H) and number of process intersections with shells at distances (in 1 μm increments) from the soma (I). (J–L) Ramification analysis (as in G–I) of microglia in perfusion-fixed P12 rats that had been anaesthetised for 1 hr either with isoflurane or urethane. p values in (I) and (L) compare distributions (using two-way ANOVA). Data are represented as mean ± SEM. See also Figures S2 and S5.

Confirming the effect of pharmacologically blocking THIK-1, microglia in hippocampal slices from THIK-1 KO mice showed a 43% reduction in surveillance index compared to littermate controls (p = 2 × 10^−18^), with heterozygote mice having intermediate values (Figures 4A and 4B; Movie S5). Plotting the time course of the increase in surveyed area in maximum intensity projections showed that the initial rate of surveillance was reduced by 41% (p = 1.2 × 10^−9^) and that the cumulative area (in maximum intensity projections) surveyed after an hour was reduced by 31% (p = 6 × 10^−7^) in the THIK-1 KO (Figures 4C and 4D; Movies S5 and S6). Carrying out a similar analysis *in vivo* on WT (Movie S7) and THIK-1 KO mice revealed a similar 45% decrease of surveillance index (p = 4.4 × 10^−8^) and a 38% reduction (p = 5.6 × 10^−9^) of area surveyed after 20 min (Figures 5A–5D; Movie S8). Thus, microglia without THIK-1 channels survey less brain volume per unit time than their WT counterparts, and so require a longer time to survey a given brain volume.

Microglia with THIK-1 knocked out also showed a much less complex ramification pattern, both in brain slices and *in vivo* (Figures 4A, 5A, and 5G; Movies S5 and S6). In perfusion-fixed mice, capturing microglial morphology as it occurs in the undisturbed brain *in vivo*, THIK-1 KO led to no change in mean microglial density in the tissue or tiling pattern (Figures 5E and 5F), but a 3D Sholl analysis of microglia revealed a significantly decreased number of processes, smaller total process length, and fewer process intersections with shells at different radii from the cell soma (Figures 5G–5I). (This differs from the situation when the CNS is repopulated with microglia, which results in a reduction of process length but an increase of cell density [Varvel et al., 2012].) The decrease in process number and ramification (Figures 5G–5I) is a major reason for the decreased surveillance seen with THIK-1 blocked or knocked out. In contrast, KO of TWIK-2 (Lloyd et al., 2011), another two-pore domain K^+^ channel expressed in microglia (see above), had no effect on microglial morphology (Figure S2).

The block of THIK-1 by isoflurane demonstrated in Figures 2C and 2E–2H and the resulting decrease in ramification and surveillance seen in Figure 3J suggest that anaesthetizing animals with isoflurane (or related gaseous anesthetics) might alter microglial properties, in contrast to using an anesthetic such as urethane, which does not affect THIK-1 (15 mM urethane reduced the mean ATP-evoked current at 0 mV, as in Figure 2C, by 4.0% ± 0.4% in 4 cells). A Sholl analysis of microglial morphology in perfusion-fixed rats that inhaled 3% isoflurane (in O_2_) for 1 hr or were instead anaesthetized with urethane (and breathed O_2_) for 1 hr revealed that the animals receiving the isoflurane displayed fewer processes and shorter total process length than those receiving the urethane (Figures 5J–5L). Thus, gaseous anesthetics can reduce microglial ramification, which will in turn reduce surveillance.

Our data show that P2Y_12_ activity is necessary for directed motility (Haynes et al., 2006), but not for surveillance. In contrast, tonic THIK-1 activity is essential for maintaining normal microglial ramification and immune surveillance of the brain but is not needed for directed motility.

### Microglial Membrane Potential Regulates Ramification and Surveillance

**Figure 6:**
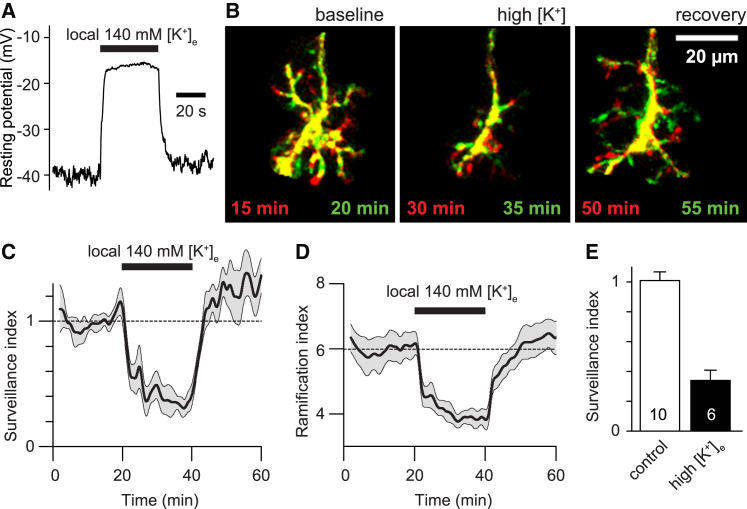
Effect of Depolarization on Microglial Ramification and Surveillance (A) Effect of locally applying 140 mM [K^+^]_o_ solution on P12 rat microglial membrane potential in hippocampal slices. (B) Images taken 5 min apart of microglial morphology in normal solution, during depolarization by perfusion of high-[K^+^]_o_ solution in TTX, and on recovery from the high-[K^+^]_o_ solution (colors as in Figure 1A). (C and D) Time course of (C) surveillance and (D) ramification during application of high-[K^+^]_o_ solution. (E) Mean surveillance index in control and high-[K^+^]_o_ solution. Data are represented as mean ± SEM. See also Figure S3.

To test whether the effect of THIK-1 on surveillance was mediated by the voltage changes that alterations of THIK-1 activity produce, we locally applied (through a patch pipette) an extracellular solution containing 140 mM [K^+^] (in the presence of 0.5 μM TTX throughout to prevent neuronal hyperactivity). This reversibly depolarized the targeted rat microglia by 23.2 ± 1.7 mV in 6 cells (Figure 6A), resulting in the microglia temporarily and reversibly retracting their processes and decreasing surveillance by 67% (p = 5.9 × 10^−5^; Figures 6B–6E), similar to the effect of the THIK-1 inhibitors and gene KO (it was not feasible to similarly investigate the effect of hyperpolarization accurately because superfusing solution lacking K^+^ produced a hyperpolarization of only 6.1 ± 0.9 mV in 6 microglia). This suggests that the rate of microglial surveillance of the brain is regulated by the cells’ membrane potential, which is controlled by the tonic level of THIK-1 activity.

Consistent with this conclusion, when 22.5 mM [K^+^]_o_ solution was superfused onto hippocampal slices from WT and THIK-1 KO mice (which depolarizes WT but not KO microglia; see above), microglial surveillance was reduced for WT microglia but was not further reduced for KO microglia (Figure S3).

### THIK-1 Regulates Interleukin-1β Release

**Figure 7:**
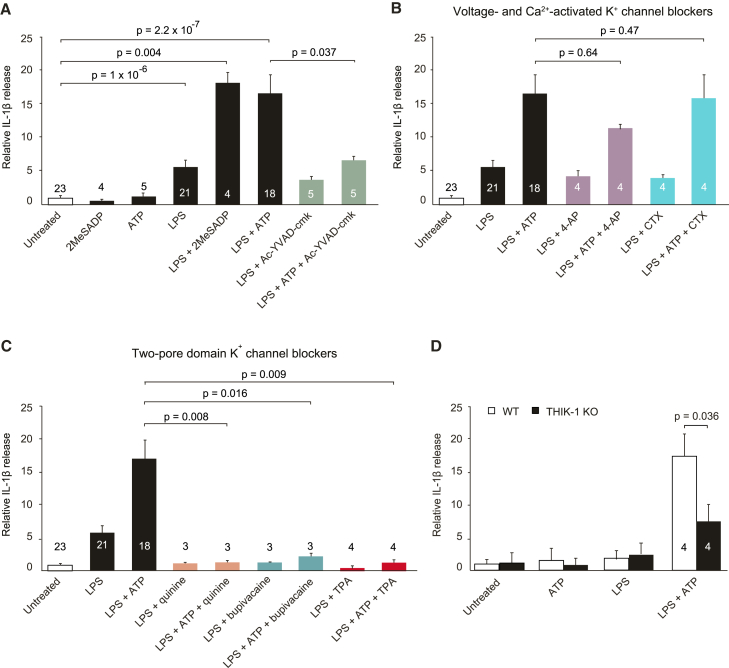
THIK-1 Inhibition Suppresses Interleukin-1β Release (A) ELISA-measured IL-1β levels released from P12 rat hippocampal slices exposed to (for durations, see STAR Methods) no drugs, the P2Y_12_ (and P2Y_13_ and P2Y_1_) receptor agonist 2-MeSADP (50 μM), ATP (1 mM), LPS (10 μg/ml), 2-MeSADP+LPS, ATP+LPS, and LPS or ATP+LPS in the presence of the caspase-1 blocker Ac-YVAD-cmk (50 μM) (numbers on bars are animals). (B) Lack of effect of voltage- and Ca^2+^-activated K^+^ channel blockers (4-aminopyridine, 4-AP, 1 mM; charybdotoxin, CTX, 1 μM) on the IL-1β release evoked as in (A). (C) Effect of two-pore domain K^+^ channel blockers (quinine, 200 μM; bupivacaine, 50 μM; tetrapentylammonium, TPA, 50 μM) on the IL-1β release evoked as in (A). Control, LPS, and LPS+ATP data from (A) are included in (B) and (C) for comparison. (D) ELISA-measured IL-1β levels released from hippocampal slices from P20-32 WT and THIK-1 KO mice in control conditions, or treated with LPS (50 μg/ml), ATP (1 mM), or ATP+LPS as in (A). Data are normalized to control data in rats (10.9 ± 3.1 pg/ml/cm^2^ of slice, n = 23 animals, with 2 slices averaged per animal) or WT mice (1.30 ± 0.78 pg/ml/cm^2^ of slice, n = 4 animals, with 2 slices averaged per animal). Data are represented as mean ± SEM. See also Figure S5.

Finally, we investigated whether THIK-1 activity was needed for the generation and release of immune mediators when microglia become activated. IL-1β is a major pro-inflammatory cytokine generated in response to infection, which contributes to tissue injury during disease. The production of IL-1β from innate immune cells such as macrophages and microglia requires the formation of inflammasome complexes to activate caspase-1, which generates interleukin-1β from its inactive precursor. Inflammasome assembly is a two-stage process involving priming by a Toll-like receptor agonist such as the bacterial coat component lipopolysaccharide, followed by a fall of intracellular [K^+^] evoked by an activating signal such as ATP (Muñoz-Planillo et al., 2013). We reasoned that K^+^ loss evoked by ATP might occur via THIK-1 in microglia. Applying only ATP (1 mM) or the P2Y_12_ (and P2Y_13_ and P2Y_1_) receptor agonist 2-MeSADP (50 μM) for 3 hr to rat hippocampal slices evoked very little interleukin-1β release into the external solution (Figure 7A). Applying lipopolysaccharide (LPS, 10 μg/ml) for 6 hr evoked some interleukin-1β release, which was greatly enhanced when P2Y_12_ receptors were activated by ATP or 2-MeSADP to increase THIK-1 activity during the last 3 hr of LPS exposure (activation by the P2Y agonist 2-MeSADP suggests that P2X_7_ receptor activity was not needed for inflammasome assembly in these experiments). Interleukin-1β release was inhibited by the caspase-1 blocker Ac-YVAD-cmk (50 μM) (Figure 7A). Blocking voltage- and Ca^2+^-gated K^+^ channels with 4-AP (1 mM) or charybdotoxin (1 μM) had no significant effect (Figure 7B), but blocking THIK-1 with quinine (200 μM), bupivacaine (50 μM), or tetrapentylammonium (50 μM) abolished the release of interleukin-1β evoked by LPS+ATP (Figure 7C). Similarly, KO of THIK-1 greatly reduced the interleukin-1β release evoked by LPS+ATP from mouse hippocampal slices (Figure 7D). Thus, THIK-1 activity is essential for the assembly of microglial inflammasome complexes and for interleukin-1β release in response to LPS+ATP.

## Discussion

**Figure 8:**
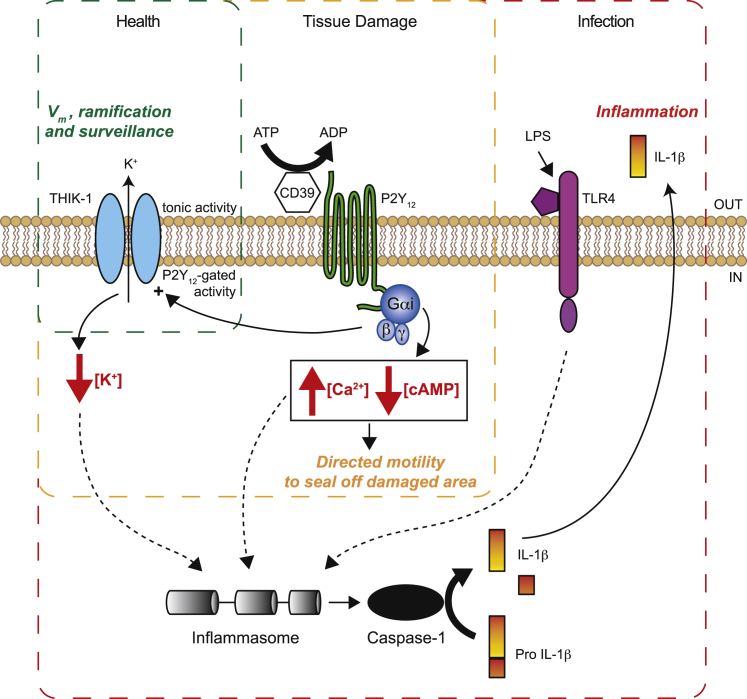
THIK-1 Regulates Microglial Ramification, Surveillance, and Interleukin-1β Release Schematic showing how THIK-1 and P2Y_12_ are central to the functions of microglia. In healthy conditions (green dashed box), tonic activity of THIK-1 maintains a negative resting membrane potential (V_m_) which is essential for normal microglial ramification and surveillance of the brain (Figure 1, Figure 2, Figure 3, Figure 4, Figure 5, Figure 6). When tissue damage occurs (orange box), ATP is released and converted to ADP by the ecto-ATPase CD39 (which we have not studied in this paper but which is believed to be an essential part of the mechanism by which ATP release leads to activation of P2Y_12_). This activates P2Y_12_, which we have shown potentiates the activity of THIK-1 (Figures 1 and 2), hyperpolarizing the membrane further. P2Y_12_ activation evokes process outgrowth to seal off the damaged area, but this does not require THIK-1 activation (Figures 3B and 3K), and so presumably reflects the other known actions of P2Y_12_, i.e., lowering [cAMP]_i_ and raising [Ca^2+^]_i_ (black box). Inflammasome assembly (red box) is triggered by the combination of activation of Toll-like receptor 4 (TLR4) by, for example, LPS—a priming stimulus—and activation of P2Y_12_ (and possibly P2Y_13_) by ATP or ADP. Loss of K^+^ from the cell and a fall of [K^+^]_i_ is needed for inflammasome assembly (Muñoz-Planillo et al., 2013), and this is mediated by THIK-1, since block or KO of this channel prevents the release of the inflammatory cytokine interleukin-1β (Figure 7).

Our data establish two functionally and mechanistically distinct modes of microglial motility (summarized in Figure 8). Directed motility to an ATP source or laser-induced tissue damage is mediated by P2Y_12_ receptors (Haynes et al., 2006) but does not require activity of the THIK-1 subunit-containing two-pore domain K^+^ channels that these receptors gate (Figures 3A, 3B, and 3K). In contrast, microglial ramification and surveillance of the brain do not require P2Y_12_ activity (or any signaling that it evokes, e.g., changes of microglial cAMP or [Ca^2+^]_i_), but are fundamentally dependent on the tonic activity of THIK-1 channels (Figures 3C–3J and 3L), which maintain the resting potential of the microglia (Figures 2D–2H and 2J). Thus, directed motility does not depend on surveillance, since THIK-1 block inhibits surveillance (Figures 3G, 3H and 3L) but does not affect directed motility (Figures 3B and 3K). Surprisingly, directed motility does not require the normal negative membrane potential that is maintained by THIK-1. However, depolarizing microglia, by blocking or knocking out THIK-1 or by locally raising [K^+^]_o_, decreases microglial ramification and inhibits surveillance (Figures 2G, 2J, 4, 5A–5D, 5G–5I, 6, and S3). This depolarization-induced decrease of ramification differs from that occurring during microglial activation (which takes hours; Kurpius et al., 2006) in that it is rapid (∼10 min) and reversible (Figure 6D). Nevertheless, it is an interesting possibility that the decreased ramification seen after activation may at least partly reflect a decrease of expression of THIK-1, since Holtman et al. (2015) found a more than 2-fold downregulation expression of mRNA for THIK-1 when cells were activated by LPS.

The process movements underlying microglial surveillance are generated by the actin cytoskeleton (Hines et al., 2009), but how THIK-1 activity and membrane hyperpolarization alter the cytoskeleton to regulate surveillance is currently unknown. Spontaneous [Ca^2+^]_i_ elevations in microglia are far too infrequent to generate surveillance movements (Pozner et al., 2015). Since directed motility is unaffected by blocking THIK-1 with tetrapentylammonium (Figures 3B and 3K), the cytoskeletal events generating directed motility in response to P2Y_12_ activation must reflect P2Y_12_-mediated signaling events independent of THIK-1 (and the K^+^ flux and voltage change it produces), such as a rise of [Ca^2+^]_i_ or a fall of [cAMP]_i_.

A combination of transcriptome (Butovsky et al., 2014, Zhang et al., 2014, Hickman et al., 2013), electrophysiological (Figures 1D–1K), pharmacological, and gene KO (Figure 2) data strongly implies that THIK-1 is the dominant component of the K^+^ channel maintaining the resting potential of microglia, and thus maintaining microglial ramification and continuous surveillance of the brain via process movement. It is generally assumed that this microglial surveillance is needed not only for the detection of pathogens and brain damage (Hanisch and Kettenmann, 2007) but also to monitor the activity of neurons and synapses, dampening activity (Li et al., 2012), and pruning synapses (Wake et al., 2009, Tremblay et al., 2010, Schafer et al., 2012) when necessary. Furthermore, we find that P2Y_12_ receptor-mediated THIK-1 activity is essential, following priming of microglia with LPS, for the release of interleukin-1β, a key pro-inflammatory driver in many neurodegenerative diseases. This process requires the K^+^-efflux-triggered assembly and activation of inflammasome complexes in immune cells (Muñoz-Planillo et al., 2013), so, as THIK-1 is the dominant K^+^ conductance in microglia, it is likely that the K^+^ efflux occurs largely via THIK-1 (Figure 8).

Interestingly, transcriptome analysis indicates that THIK-1 is not expressed in the cultured microglia often used to assess CNS immune cell function or in macrophages or other immune cells (Butovsky et al., 2014), which explains why its role has not previously been identified. Identification of the importance of THIK-1 will allow experiments modulating it to assess the contribution of microglial surveillance to the diverse functions that these cells are thought to carry out, including synaptic pruning, modulating neuronal firing, detecting pathogens, and phagocytosing dead neurons.

THIK-1 expression may vary between microglia in different CNS locations, contributing to differences in microglial morphology, surveillance, and release of immune mediators. In the basal ganglia, microglia in the substantia nigra pars reticulata have a resting potential that is on average 10–20 mV more negative than the resting potential of microglia in the substantia nigra pars compacta or the ventral tegmental area, and pars reticulata microglia also exhibit a considerably more ramified shape (De Biase et al., 2017). Given the effect of membrane potential on microglial morphology and surveillance that we observe (Figures 6 and S3), these data are consistent with the idea that pars reticulata microglia express more THIK-1 (relative to other membrane ion channels) than the microglia in nearby areas. In future work it will be interesting to make a mouse in which it is possible to optogenetically change microglial membrane potential and examine the effect in different brain areas on ramification, surveillance, and release of immune mediators.

THIK-1 mRNA expression increases after microglia enter the brain around embryonic day 13.5 (Matcovitch-Natan et al., 2016) and then is approximately constant until adulthood, but then decreases with age (Hickman et al., 2013), which may impair CNS immune surveillance and inflammatory responses in the elderly. Similarly, gaseous anesthetics inhibit THIK-1 (Figures 2C and 2E–2H) and may suppress microglial function during operations under anesthesia. The inhibitory effect of isoflurane anesthesia on microglial ramification and surveillance, both in slices (Figures 3I, 3J, and 3L) and *in vivo* (Figures 5J–5L), suggests that future *in vivo* experiments studying motility should avoid the use of isoflurane or other anesthetics that block THIK-1. The increase of extracellular potassium concentration to ∼60 mM during the spreading depression associated with migraine, stroke, sub-arachnoid hemorrhage or brain injury (Lauritzen et al., 2011), or the anoxic depolarization induced by ischemia (Hansen, 1985), and the 10 mM [K^+^]_o_ rise occurring during synchronous high-frequency activity of many neurons as occurs in epilepsy (Hansen, 1985), are also expected to depolarize microglia and decrease their ramification and surveillance (Figures 6 and S3). In contrast, the [K^+^]_o_ rise occurring during normal neuronal activity (∼1 mM; Hansen, 1985) is much smaller and will have little effect.

The impairment of motility of microglial processes that occurs in some pathological conditions, e.g., in models of Alzheimer’s disease with amyloid β plaque deposition (Koenigsknecht-Talboo et al., 2008, Krabbe et al., 2013, Condello et al., 2015) raises the question of whether the dependence of surveillance on THIK-1 activity can be employed therapeutically. Increasing THIK-1 activity might enhance surveillance and neuroprotection (in disease or old age) or increase synaptic pruning during development (e.g., to reduce changes leading to autism; Zhan et al., 2014), while decreasing THIK-1 activity could be employed to reduce these microglial actions (e.g., to reduce microglial-mediated damage to bystander neurons in disease; Bialas et al., 2017).

## STAR★Methods

### Key Resources Table

**Table:** 

REAGENT or RESOURCE	SOURCE	IDENTIFIER
**Antibodies**		

Iba1	Synaptic systems	Cat#234003
Donkey anti-rabbit IgG (H+L) Alexa Fluor 488-congugated secondary	ThermoFisher	Cat#A-21206
Donkey anti-rabbit IgG (H+L) Alexa Fluor 555-congugated secondary	ThermoFisher	Cat#A-31572

**Chemicals, Peptides, and Recombinant Proteins**		

Lipopolysaccharide	Sigma	*Escherichia coli* 055:B5, Cat#L2880
DMEM	GIBCO	Cat#41965-039
MEM	GIBCO	Cat#42360-032
ATP-Mg salt	Sigma-Aldrich	Cat#A9187
ATP-Na salt	Sigma-Aldrich	Cat#A7699
ADP	Sigma-Aldrich	Cat#A2754
Adenosine	Sigma-Aldrich	Cat#A9251
NEM	Sigma-Aldrich	Cat#E3876
GDPβS	Sigma-Aldrich	Cat#G7637
Pertussis toxin	Invitrogen	Cat#PHZ 1174
2-MeS-AMP	Tocris	Cat#1624
MRS-2211	Tocris	Cat#2402
PSB-0739	Tocris	Cat#3983
4-aminopyridine	Sigma-Aldrich	Cat#A78403
Margatoxin	Alomone	Cat#STM-325
Charybdotoxin	Anorspec	Cat#28244/ Cat#22428
Paxilline	Tocris	Cat#2006
Quinidine	Sigma-Aldrich	Cat#Q3625
Quinine	Sigma-Aldrich	Cat#Q1125
Bupivacaine	Sigma-Aldrich	Cat#B5274
Tetrapentylammonium	Sigma-Aldrich	Cat#258962
Propafenone	Sigma-Aldrich	Cat#P4670
Lamotrigine	Tocris	Cat#2289
Halothane	Merial	Cat#B07707A
Isoflurane	Abbott	Cat#B506
Sevoflurane	Abbott	Cat#4456
HgCl_2_	Sigma-Aldrich	215465
Urethane	Sigma-Aldrich	Cat#94300
TTX	Cayman	Cat#14964
IsolectinB4-Alexa594	Invitrogen	Cat#121413
IsolectinB4-Alexa488	Invitrogen	Cat#121411
Ac-YVAD-cmk	Sigma-Aldrich	Cat#SML0429

**Critical Commercial Assays**		

ELISA for rat interleukin-1β	R&D Systems	RLB00
ELISA for mouse interleukin-1β	R&D Systems	MLB00C

**Experimental Models: Organisms/Strains**		

THIK-1 KO mouse	MRC Harwell	Kcnk13-INS1-EM1-B6N
Iba1-GFP mouse	Prof S Kohsaka	Iba1-GFP mouse
TWIK-2 KO mouse	Prof RM Bryan	TWIK-2 KO mouse

**Software and Algorithms**		

ImageJ	NIH	https://fiji.sc/ or https://imagej.nih.gov/ij/
Vaa3D	Allen Institute	http://www.alleninstitute.org/what-we-do/brain-science/research/products-tools/vaa3d/
Directed Motility Analysis software	Written in house	N/A
Surveillance Analysis software	Written in house	N/A
Ramification Analysis software	Written in house	N/A
Sholl Analysis software	Written in house	N/A

### Contact for Reagent and Resource Sharing

Requests for further information, resources, and reagents should be directed to and will be fulfilled by the Lead Contact, David Attwell (d.attwell@ucl.ac.uk). Iba1-GFP mice are subject to restrictions imposed in an MTA by S. Kohsaka. THIK-1 KO mice are subject to restrictions imposed in an MTA by the Medical Research Council, UK.

### Experimental Model and Subject Details

Experiments used Sprague-Dawley rats, transgenic mice expressing eGFP under control of the Iba1 promoter (Hirasawa et al., 2005), or transgenic mice with THIK-1 expression deleted (see below), of either sex. Pre-weaning animals were housed with their mother and sometimes the father as well; weaned animals were housed in groups of 2-5. Housing was in individually ventilated cages. Animal procedures were carried out in accordance with the guidelines of the UK Animals (Scientific Procedures) Act 1986 and subsequent amendments. All data are from rats except where mice are explicitly stated. Ages are stated in the figure legends for each experiment.

### Method Details

#### Brain slice preparation

Acute hippocampal slices (300 μm thick) were prepared (Bischofberger et al., 2006) from P12 Sprague-Dawley rats or P15-P27 (as stated in the text) transgenic mice in ice-cold solution containing (mM) 124 NaCl, 26 NaHCO_3_, 1 NaH_2_PO_4_, 2.5 KCl, 2 MgCl_2_, 2 CaCl_2_, 10 glucose, bubbled with 95% O_2_/5% CO_2_, pH 7.4, as well as 1 mM Na-kynurenate to block glutamate receptors. Brain slicing does not activate microglia for at least 4 hours, as judged by cell morphology, motility and interleukin 1β release (Kurpius et al., 2006, Gyoneva and Traynelis, 2013), and allows pharmacological investigation of mechanisms in a manner that is not possible using *in vivo* experiments. Slices were incubated in darkness at room temperature (22–24°C) in oxygenated HEPES-buffered external solution (see below) containing 25 μg/ml Alexa 594 conjugated isolectin B_4_ for all experiments involving imaging (or imaging and electrophysiology) or 25 μg/ml Alexa 568 conjugated isolectin B_4_ (for electrophysiology experiments without imaging) for 30 min (Kurpius et al., 2006, Grinberg et al., 2011) before being used in experiments. Isolectin B_4_ labeling does not activate microglia (Grinberg et al., 2011) and avoids function changes which might occur in transgenically-labeled microglia; however, the microglial labeling is somewhat dimmer than in the Iba1-GFP mice.

#### External solutions

Slices were superfused with HEPES-buffered solution, at 34-36°C for all experiments involving imaging (or imaging and electrophysiology) and at room temperature (22-24°C) for purely electrophysiological experiments, containing (mM) 140 NaCl, 2.5 KCl, 10 HEPES, 1 NaH_2_PO_4_, 2 CaCl_2_, 1 MgCl_2_, 10 glucose, pH set to 7.4 with NaOH, bubbled with 100% O_2_. For experiments involving application of K^+^ channel blockers 0.5 μΜ TTX was added to all external solutions to prevent any changes in neuronal activity that might occur.

#### Intracellular solutions

Cells were whole-cell clamped with electrodes containing KCl (or CsCl for Figure 1G) based solution, comprising (mM) 125 KCl (or CsCl), 4 NaCl, 1 CaCl_2_, 10 HEPES, 10 EGTA, 4 MgATP, 0.5 Na_2_GTP, pH set to 7.1 with KOH or CsOH. Final osmolarity was 285 ± 5 mOsm.

#### Electrophysiology

Microglia were identified by their fluorescent label and ramified morphology, and whole-cell clamped at a depth of ∼50-100 μm in the slice at room temperature (22-24°C), or at 34-36°C if combined with imaging or laser damage experiments, using borosilicate pipettes with a tip resistance of ∼5 MΩ, giving a series resistance of < 20 MΩ. Electrode junction potentials were compensated. I-V relations were from responses to 200 msec voltage steps.

#### Two-photon imaging and laser-evoked tissue damage

Microglia in hippocampal slices were imaged at 34-36°C, at a depth of ∼50-100 μm in the slice (to avoid studying superficial microglia that had started to become activated by the slicing procedure) using a Zeiss LSM 710 or 780 microscope (with a 20X lens, NA 1.0) and a Spectraphysics Mai Tai DeepSee eHP Ti:sapphire infrared laser. For imaging of microglia labeled with isolectin B_4_-Alexa 594, the laser was tuned to a wavelength of 800 nm, while for imaging cells labeled with eGFP a wavelength of 920 nm was used; generally the laser was adjusted to 1.8% of its maximum power at 800 nm or 6%–8% at 920 nm, corresponding to ∼5 mW and ∼12 mW respectively at the preparation, i.e., well within the intensities used by others (Wake et al., 2009, Hines et al., 2009, Pfeiffer et al., 2016). The pixel dwell time was 1 μsec. Ablation of a small volume of tissue (“laser damage”) was performed by illuminating a ∼5 μm radius spot with the laser intensity increased 30-fold and the pixel dwell time increased to 100 μsec. For imaging of directed process motility in response to a pipette filled with 1 mM ATP or laser-induced tissue damage, stacks of 21-31 slices imaged at 2 μm depth intervals were acquired every 30 s. For imaging of microglial surveillance in the absence of damage or applied ATP (baseline surveillance), stacks of 21-31 slices imaged at 2 μm depth intervals were acquired every 60 s. Images were typically 512 by 512 pixels and covered a square field of view 200 to 250 μm wide.

#### Analysis of imaging data

Analysis of two-photon images was performed using custom-written ImageJ (NIH) and MATLAB scripts (The MathWorks). The software is available on request.

For analysis and quantification of microglial directed process motility in response to a pipette filled with 1 mM ATP or laser-induced tissue damage, each slice of every stack was first filtered with a median filter (which replaces each pixel value with the median value of the 3 pixel x 3 pixel array centered on that pixel). We then performed a maximum intensity projection, registered (Thévenaz et al., 1998), and binarised the resulting two-dimensional movies (setting the threshold for binarisation manually). The resulting movies were processed using a MATLAB script inspired by the algorithm described by Gyoneva et al. (2014). Briefly, after the user manually clicks on the final target of chemotactic processes (i.e., either the tip of the glass pipette containing ATP or the center of the area damaged with the laser), the algorithm divides the surrounding area into concentric circles with radii at 2 μm intervals, and then segments these circles into 32 radial sectors, thus creating 32 patches between every two consecutive concentric circles. Then, for each frame, starting from the center, the algorithm searches in every radial sector for the first patch containing > 10 positive pixels (labeled microglia). The outputs of the algorithm are, for each frame, (i) the distance to the microglial process front in each sector and (ii) the surface area contained within the converging microglial process front.

For analysis and quantification of microglial surveillance, each slice of every stack was filtered with a median filter after subtraction of smooth continuous background with the ImageJ ‘subtract background’ plugin with a ball size of 30 pixels. The 4D stacks were then registered first for lateral drift, then rotated 90° on their side, registered for z-drift and rotated back to their original orientation. We then performed a maximum intensity projection. Cells of interest were individually selected by manually drawing a region of interest (ROI) around an area including all their process extensions over the whole duration of the resulting 2D movie and erasing data around that ROI. These 2D movies of individual cells were then manually binarised and saved as independent files.

To quantify ramification, the resulting movies were processed using MATLAB. In each movie frame, the MATLAB functions bwarea (www.mathworks.com/help/images/ref/bwarea.html) and bwperim (http://mathworks.com/help/images/ref/bwperim.html) with 8-connected neighborhood were used to quantify respectively the area and the perimeter of the cell. The ramification index *R* is defined as the ratio of the perimeter to the area, normalized by that same ratio calculated for a circle of the same area. Specifically:

$$\mathrm{R=}\left(\text{perimeter}/\text{area}\right)/\left[2\text{.}{\left(\pi /\text{area}\right)}^{1/2}\right].$$

Thus, *R* = 1 if the cell is a perfect circle. The more ramified the cell, the larger *R* is. In 134 cells in hippocampal slices the mean value of *R* was 6.44 ± 0.14 at the beginning of the experiment, implying that the cells have a 6.44-fold larger circumference than a circle of the same area. Scaling up the cell size, to (say) double its area, would have no effect on the ramification index. Ramification indices plotted in Figures 3 and 6 are averaged over all cells for a specific condition.

To quantify surveillance, for each movie, starting with the second frame, we subtracted from each binarised frame *F*_t_ the preceding frame *F*_t-1_ and created two binarised movies, *PE* consisting of only the pixels containing process extensions (*F*_t_-*F*_t-1_ > 0) and *PR* consisting of only the pixels containing process retractions (*F*_t_-*F*_t-1_ < 0). In both *PE* and *PR*, all other pixels are set to 0. The baseline surveillance index *B* is defined as the sum over all non-zero pixels in *PE* + *PR* normalized by its average over the initial 20 minutes of each experiment, i.e.:

$$B={\left(\sum_{\text{pixels}}\mathrm{PE+PR}\right)/\langle \left(\sum_{\text{pixels}}\mathrm{PE+PR}\right)\rangle }_{\text{control}}$$

where Σ_pixels_ denotes a sum taken over all non-zero pixels and < >_control_ denotes a temporal average taken over the control period of the experiment (the first twenty minutes here). Surveillance indices for WT, THIK-1 heterozygote and THIK-1 KO mice were calculated as absolute values according to *B* = (Σ_pixels_ *PE*+*PR*) for each genotype, and subsequently normalized to the WT condition. The surveillance index provides a measure of the brain volume that is surveyed by a microglial cell in a given time. It is affected both by the rate at which processes elongate and shorten, and by the overall number of the microglial processes and their mean length. Thus, when THIK-1 is blocked or knocked out and microglia depolarize, the resulting deramification of the cells and shortening of their total process length (Figures 3G–3J, 3L, 4, 5, and 6) will reduce the volume of brain that is surveyed, and this is reflected in the surveillance index. Although overall surveillance will also, in principle, be affected by the density of microglia, this is unaffected either by short-term drug application or by KO of THIK-1 (Figures 5E and 5F).

Although some unavoidable bleaching of the microglial label occurred during the prolonged imaging used for this work, this did not affect the derived values of surveillance index, because our analysis of surveillance is carried out on binarised images and so is unaffected by bleaching provided that processes are still well resolved, as they were in our movies. Indeed, prolonged imaging did not reduce the surveillance or ramification of the microglia when assessed over 60 mins of imaging (Figure 3D), and the less ramified structure of microglia lacking THIK-1, compared to WT microglia, was seen even in perfusion-fixed tissue where the microglia had not been exposed to laser illumination (Figures 5G–5I; Movie S6).

#### Microglial immunostaining and Sholl analysis

Microglial morphology was assessed by Sholl analysis (Sholl, 1953). To compare changes in microglial morphology produced by deletion of THIK-1 expression, 3 THIK-1 KO and 3 control WT mice were killed at P17-21 by sodium-pentobarbital overdose and fixed by transcardial perfusion of 4% paraformaldehyde (PFA). Brains were then removed and post-fixed for 24 hr in 4% PFA. To compare changes in microglial morphology produced by deletion of TWIK-2 expression, 3 TWIK-2 KO and 3 control WT mice (at P19-22) were given i.p. injections of heparin and of ketamine/xylazine/acepromazine anesthetic, and then transcardially perfused with 10% neutral buffered formalin (NBF, 4% formaldehyde). Brains were then fixed overnight in 10% NBF before being cryoprotected in 30% sucrose/PBS and frozen at −80°C until slicing. To compare changes in microglial morphology produced by different anesthesia protocols, P12 rats were anaesthetised with an i.p. injection of urethane (1.55 g/kg; Sigma-Aldrich, 3 animals) and ventilated with O_2_ (2 l/min), or anaesthetised by inhaling 3% isoflurane mixed with O_2_ (2 l/min, 3 animals) for 1 hour and fixed by transcardial perfusion of 4% PFA. Brains were removed and post-fixed for 24 hr. Horizontal sections (60-75 μm, “magic cut,” Bischofberger et al., 2006) were cut on a vibratome (Leica). Tissue sections were blocked and permeabilised in 10% horse serum and 0.5% Triton X-100 in PBS for 1 hour at room temperature. Slices were incubated at 4°C overnight in rabbit anti-Iba1 (Synaptic Systems 234003) antibody prepared in blocking solution. After washing 3 times for 20 mins in PBS, slices were incubated overnight in secondary antibody (donkey anti-rabbit Alexa Fluor 488 or Alexa Fluor 555, Invitrogen). Slices were then washed in PBS, incubated in DAPI (Invitrogen) and mounted on glass slides using DAKO fluorescence mounting medium. All images were acquired using a 63X oil immersion objective (NA 1.4) on a Zeiss LSM700 confocal microscope. All analysis was carried out with the experimenter blinded to the genotype. Cell reconstructions were carried out using 3D automatic cell tracing in Vaa3D software (http://home.penglab.com/proj/vaa3d/). Morphological parameters were then extracted using custom code written in MATLAB.

#### Measurement of anesthetic concentration for slice experiments

The gaseous anesthetics were prepared as saturated solutions (concentrations 17.8 mM for halothane, 15.3 mM for isoflurane and 11.8 mM for sevoflurane), and were diluted to give lower concentrations in some experiments. The anesthetics are partly lost through the walls of the perfusion tubes so, to determine the actual concentration reaching the slice, we collected the solution entering the recording chamber and subjected it to gas chromatography/mass spectroscopy, using known concentrations for calibration, as described by McDougall et al. (2008). Bath samples were collected and dissolved in 100% acetonitrile with a known concentration of a calibration standard (4 mM halothane for isoflurane and sevoflurane samples; 4 mM sevoflurane for halothane samples). The ratio of the area under the peak (AUP) detected in GC-MS for the tested anesthetic, to the AUP of the calibration standard, was then compared to calibration curves (Figure S4).

#### *In vivo* experiments

Microglia were imaged *in vivo* in adult transgenic mice in which eGFP was expressed under control of the Iba1 promoter (Hirasawa et al., 2005). Briefly, adult animals were anaesthetized with an i.p. injection of urethane (1.55 g/kg; Sigma-Aldrich), which does not affect the ATP-evoked THIK-1-mediated current (tested at 15 mM in 4 cells, reduced by 4.0 ± 0.4%). After we confirmed that the animals were deeply anaesthetised, we quickly shaved their heads and fixed them in a head holder (Narishige). Throughout surgery and experiment, the animals’ temperature was kept at 37°C with a heating blanket (CWE). Eyes were protected from drying by regular application of polyacrylic acid eye drops (Dr. Winzer Pharma). After topical application of lidocaine (AstraZeneca), the skull above the barrel cortex was exposed after a midline incision and disconnection of the temporal muscle from the skull. A custom made headplate with a central circular hole was then glued to the skull with a small drop of cyanoacrylate superglue. After the glue had dried, we applied dental cement (Paladur; Heraeus) to improve the fixation of the headplate and seal the circular hole so as to create a well for imaging. After the dental cement had dried, we transferred the animal to a stage custom-made to accommodate the headplate. Using a dental drill (Saeshin Precision Co.), we then carefully performed a small craniotomy ∼2-3 mm in diameter and the dura was removed above the barrel cortex. Health of the tissue was assessed during two-photon imaging by the presence of ramified microglia (Movie S7), continual process movement (Movie S8) and absence of honeycomb or jellyfish microglia (Roth et al., 2014), which we never observed. The animal was then transferred to a Zeiss LSM 710 or 780 two-photon microscope for imaging. Microglia were imaged in the barrel cortex as described above, 150-200 μm below the surface (layer 2/3), under urethane anesthesia.

#### ELISA measurements of interleukin-1β release

As previously described (Charolidi et al., 2015), hippocampal slices (300 μm thick) were prepared in ice-cold HEPES-buffered medium (MEM bubbled with O_2_, pH 7.4, 42360-032, GIBCO) under sterile conditions. To induce inflammasome activation and IL-1β release, slices were exposed to inflammatory-like stimuli (Bernardino et al., 2008). Each slice was placed on a Millicell cell culture insert (12 μm pore size, PIXP01250, Merck Millipore) and transferred into 24-well plates containing 800 μL serum-free medium (DMEM, pH 7.4, 41965-039, GIBCO) with or without lipopolysaccharide (LPS) (10 μg/ml, *Escherichia coli* 055:B5, L2880, Sigma-Aldrich) and/or potassium channel inhibitors in a cell culture incubator at 37°C. After 30 min, the medium above the slices was removed and slices were kept for 6 h in 350 μL DMEM with or without LPS and/or the K^+^ channel inhibitors, for the last 3 h of which 1 mM ATP or 50 μM 2-MeSADP was added (or not), as indicated. The concentrations of LPS used were chosen to evoke reliably detectable release of IL-1β, and are similar to those that have been described in the literature to induce inflammatory responses for both rats (Guerra et al., 2011) and mice (Hines et al., 2013); the higher concentration needed for mouse (50 μg/ml) than for rat (10 μg/ml) may reflect a species difference or a lower sensitivity of the mouse assay (Schmidt et al., 2012). The amount of IL-1β released into the medium was measured by ELISA, using Quantikine IL-1 beta/IL-1F2 kits (R&D Systems, RLB00 for rat and MLB00C for mouse). Data were from at least 3 rats or mice per experiment, from each of which 2 brain slices were used per experimental condition. Immediately after collecting the media, photographs of slices were taken and the slice surface area was determined using ImageJ. To compare data between brain slices in different conditions, the amounts of IL-1β released into the medium were normalized to the slice surface area. Rat and mouse data were then further normalized to the mean of the control values obtained in rats or in WT mice, respectively.

#### Generation of THIK-1 knock-out mice

The gene for THIK-1 (*knck13*), also known as K_2P_13.1, was disrupted in mice (by MRC-Harwell: Brown and Moore, 2012, Bradley et al., 2012, Pettitt et al., 2009, using CRISPR/Cas9 to insert a single nucleotide into the wild-type DNA sequence. This introduces a frameshift mutation into the codon for amino acid 14, leading to an abolition of the hydrophobic structure that normally forms the 1st transmembrane segment of THIK-1, and to a premature stop codon being generated after amino acid 68 in the open reading frame (Figure S5). Knock-out mice were bred from heterozygotes, which appeared to have no obvious phenotype. Microglia in the mice were visualized either by isolectin B_4_ labeling as for rats, or by crossing heterozygotes with Iba1-GFP mice to introduce GFP labeling into microglia in the KO. All surveillance imaging was done using Iba1-GFP labeled mice.

Single-guide RNA (sgRNA) Kcnk13_#5.1 was selected employing the Zhang Lab CRISPR design tool (http://crispr.mit.edu/) in order to have no potential off-target sites with < 3 mismatches and having the minimal total number of potential off-target sites (especially on the targeted chromosome 12). Off-target sites associated with sgRNA_Kcnk13_#5.1 can be found at: http://www.sanger.ac.uk/htgt/wge/crispr/355997724.

sgRNA Kcnk13_#5.1 was *in vitro* transcribed from a gBlock gene fragment (Integrated DNA Technologies) containing the following sequence (20 nucleotide protospacer sequence underlined):

GGTGTAAACCTTAAACTGCCGTACGTATAGGCTGCGCAACTGTTGGGAAGGGCGATCGGTGCGGGCCTCTTCGCTATTACGCCAGCTGGCGAAAGGGGGATGTGCTGCAAGGCGATTAAGTTGGGTAACGCCAGGGTTTTCCCAGTCACGACGTTGTAAAACGACGGCCAGTGAATTGTAATACGACTCACTATAGG*AGCGCGCGTTGTCCTCGTTC*GTTTTAGAGCTAGAAATAGCAAGTTAAAATAAGGCTAGTCCGTTATCAACTTGAAAAAGTGGCACCGAGTCGGTGCTTTTTTT.

sgRNA was synthesized using MEGAshortscript (Ambion) and purified using MEGAclear kit (Ambion). Cas9 mRNA was commercially sourced (Cas9 mRNA (5meC, Psi), Tebu-Bio).

Pronuclear microinjection was performed as previously described (Gardiner and Teboul, 2009). Injected embryos were re-implanted in CD1 pseudo-pregnant females which were allowed to litter and rear F0 progeny. The mutation was confirmed by DNA sequencing.

The single nucleotide insertion in the mutant resulted in the formation of a TaqI restriction site (Figure S5) which was used to identify the genotype of the mice used. In brief, genomic DNA was extracted from ear clip biopsies. The targeted region was PCR amplified using the following primers: AAGGTCGGCAGAGCACATC and CTGGTGGCTTCCTCGTAGTG (Figure S5) and following purification the amplified DNA was digested with TaqI at 65°C for 3hrs with the resulting product run on a 2% agarose gel. Wild-type animals displayed a single band of 330bp whereas homozygote mutants displayed 2 bands (208bp and 122bp). Heterozygotes displayed both the wild-type and mutant bands (330, 208 and 122bp; Figure S5).

### Quantification and Statistical Analysis

Data are presented as mean ± SEM. Where appropriate, drug effects were compared with bracketing control values, and experiments on mice of different genotype were interleaved. The experimenter was blind to the genotype. P values are from two tailed Student’s t tests (for normally distributed data) or Mann-Whitney U tests (for non-normally distributed data), and are given on the figures, in their legends or in the main text. Normality of data was checked using the Kolmogorov-Smirnoff or Shapiro-Wilk test and equality of variance confirmed using the F-test. P values quoted in the text are from independent samples t tests unless otherwise stated. For multiple comparisons, p values are corrected using a procedure equivalent to the Holm-Bonferroni method (for N comparisons, the most significant p value is multiplied by N, the 2nd most significant by N-1, the 3rd most significant by N-2, etc.; corrected p values are significant if they are less than 0.05). An estimate of the sample size needed for a typical experiment is as follows: For a control response of 100%, a typical response standard deviation of 25%, a response in a drug of 50% (50% inhibition), a power of 80% and p < 0.05, 6 cells are needed (http://www.biomath.info/power/ttest.htm) in each of the control and drug groups. The exact numbers vary between experiments, depending on the drug effect size and standard error of the data. Numbers of animals and cells studied are stated for each experiment in the text.

### Data and Software Availability

The code used for ramification, surveillance and Sholl analysis is available from https://github.com/AttwellLab/Microglia.

## Supporting Citations

The following reference appears in the Supplemental Information: De Simoni et al. (2008).

## Supplemental Information

Document S1. Figures S1–S5

Movie S1. Microglial Surveillance, Related to Figure 1 Surveillance of the CNS by microglia in a rat hippocampal slice. Left panel of triptych: Process movements of isolectin B_4_-Alexa 594-labeled microglial cell (maximum intensity projection). Middle: Binarized version of left panel. Right: Cumulative plot, with each pixel remaining white after a microglial process has entered it, showing how the cell gradually scans the brain volume.

Movie S2. Directed Motility of Microglia, Related to Figure 1 (Part 1) Directed motility: Convergence of microglial processes (labeled with isolectin B_4_-Alexa 594, which also labels blood vessels) in a rat hippocampal slice on an ∼10 μm diameter area (white circle) which has been ablated by the infrared laser (at the time the circle appears). (Part 2) Directed motility: Convergence of microglial processes in a rat hippocampal slice on the tip of a pipette filled with 1 mM ATP (and green Alexa 488) in external solution.

Movie S3. Pharmacology of Directed Motility, Related to Figure 3 Left: Suppressive effect of PSB-0739 (5 μM) on the directed motility of microglial processes (in a rat hippocampal slice) toward a pipette filled with 1 mM ATP in external solution (center of the grid; concentric circles at 2 μm intervals). Polygon connects microglia closest to the pipette tip (see STAR Methods and Gyoneva et al., 2014). Compare with right movie. Right: Lack of effect of tetrapentylammonium (50 μM) on the directed motility of microglial processes (in a rat hippocampal slice) toward a pipette filled with 1 mM ATP in external solution (center of the grid; concentric circles at 2 μm intervals).

Movie S4. Pharmacology of Surveillance, Related to Figure 3 Left: Lack of effect of PSB-0739 (2 μM) on microglial ramification and surveillance of the CNS (microglia labeled with isolectin B_4_-Alexa 594) in a rat hippocampal slice. Right: Suppressive effect of tetrapentylammonium (50 μM) on microglial ramification and surveillance (microglia labeled with isolectin B_4_-Alexa 594) in a rat hippocampal slice.

Movie S5. THIK-1 Maintains Microglial Surveillance, Related to Figure 4 Surveillance of the CNS by microglia in hippocampal slices from P27 WT and P21 THIK-1 KO mice, shown as maximum-intensity projections in the tryptich format of Movie S1. Graphs on the right show the time course of the cumulative pixels surveyed (as in Figures 4C and S1A) and the surveillance index (normalized to the mean value for the WT cell).

Movie S6. THIK-1 Maintains Microglial Ramification, Related to Figure 5 3D images of hippocampal microglia from perfusion-fixed P17 WT and P19 THIK-1 KO mice show much less complex process arbors in the KO.

Movie S7. Microglia *In Vivo*, Related to Figure 5 Ramified microglia in the adult Iba-1-GFP mouse somatosensory cortex imaged *in vivo*, from the cortical surface to ∼400 μm depth.

Movie S8. Surveillance by Microglia *In Vivo*, Related to Figure 5 Surveillance of the CNS by microglia *in vivo* in P23 WT and P22 THIK-1 KO mice, shown as maximum-intensity projections in the tryptich format of Movies S1 and S5. Graphs on the right show the time course of the cumulative pixels surveyed (as in Figures 4C, 5C, and S1A) and the surveillance index (normalized to the mean value for the WT cell).

Document S2. Article plus Supplemental Information
