# Supplementary material for: Microglial Ramification, Surveillance, and Interleukin-1β Release Are Regulated by the Two-Pore Domain K+ Channel THIK-1
Source: Neuron. 2018 Jan 17;97(2):299–312.e6. doi: 10.1016/j.neuron.2017.12.002 (PMC5783715; doi:10.1016/j.neuron.2017.12.002)
Supplement: Document S1. Figures S1–S5 [file mmc1.pdf]

Neuron, Volume *97*

Supplemental Information

Microglial Ramification, Surveillance, and Interleukin-1β Release Are Regulated by the Two-Pore Domain K^+ Channel THIK-1

Christian Madry, Vasiliki Kyrargyri, I. Lorena Arancibia-Cárcamo, Renaud Jolivet, Shinichi Kohsaka, Robert M. Bryan, and David Attwell

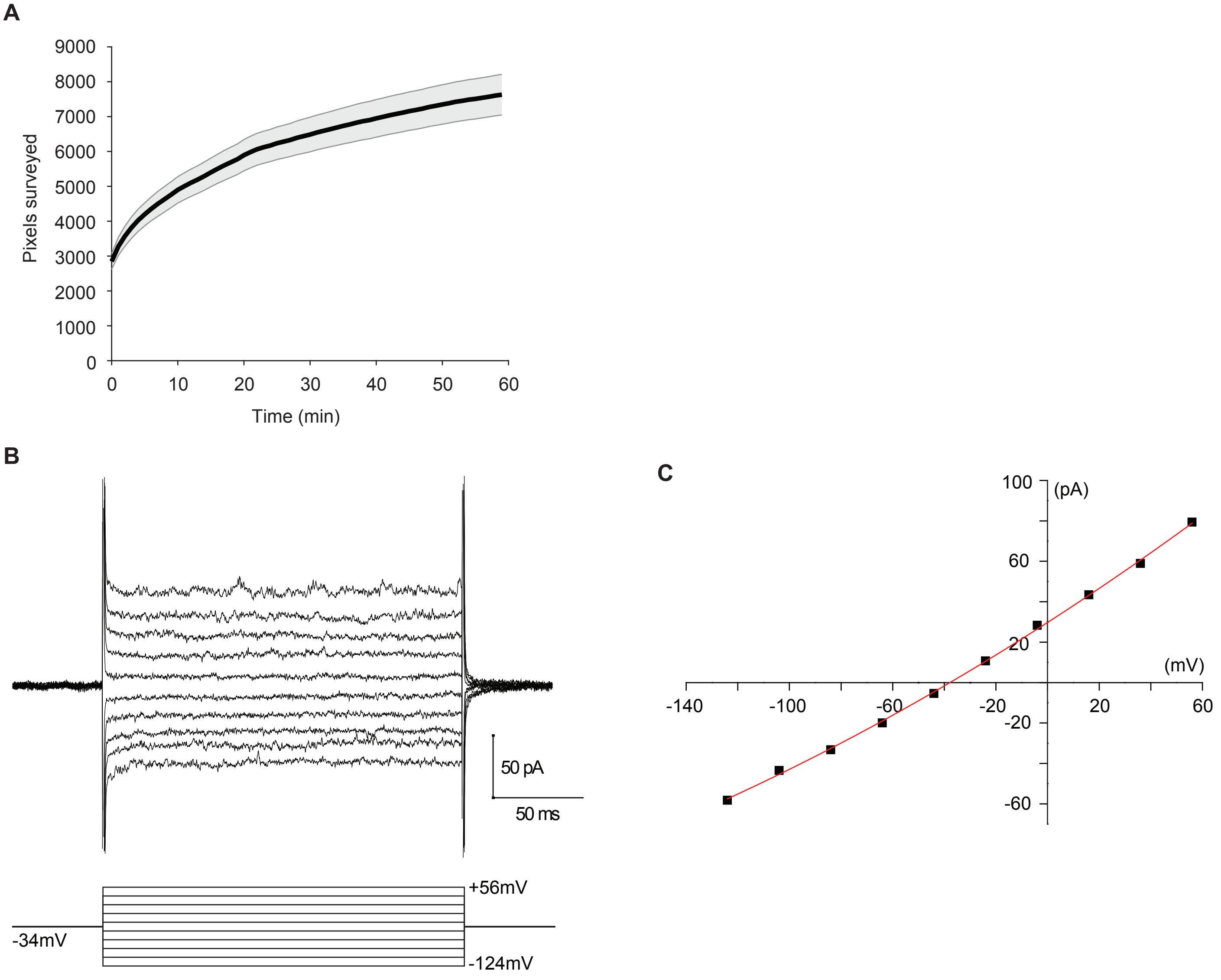

Supplementary Figure 1.

Cumulative surveillance and I-V relation of ramified microglia (related to Figures 1, 2 & 3).

(A) Time course of increase of the number of surveyed pixels (mean±s.e.m.) in maximum intensity projections of images of 10 microglia (as in Suppl. Movies 1 and 5) in rat hippocampal slices. (B) Typical current response of a ramified P12 rat microglial cell to voltage steps in 20 mV increments from -134 to +56 mV (from a holding potential of -34 mV) showing no activation of voltage-gated channels such as Kv1.3 at positive potentials, indicating that the cell is not activated (De Simoni et al., 2008). (C) Steady state I-V relation from B.

Fig. S1

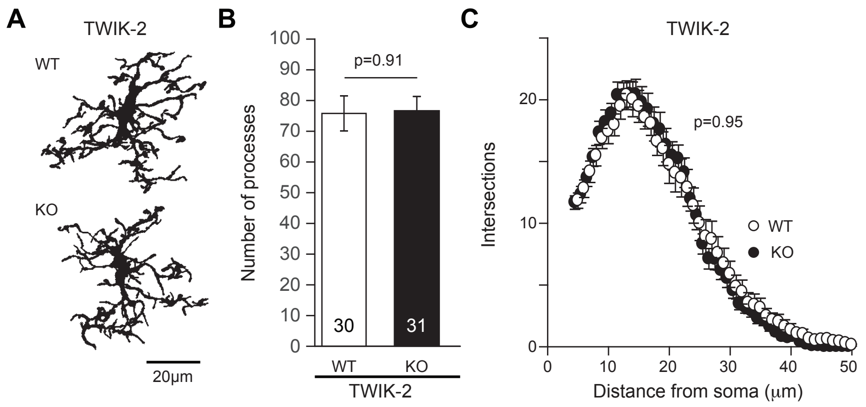

Supplementary Figure 2.

Knock-out of TWIK-2 does not affect microglial morphology (related to Figures 4 and 5).

Ramification analysis of microglia (numbers of cells on bars in B) from perfusion-fixed WT and TWIK-2 KO mice (3 of each, aged P19-22) showing (A) representative 3D-reconstructed WT and KO microglia, and (B-C) Sholl analysis derived number of processes (B) and number of process intersections with shells at distances (in 1 μm increments) from the soma (C). P value in C compares distributions (using 2-way ANOVA).

Fig. S2

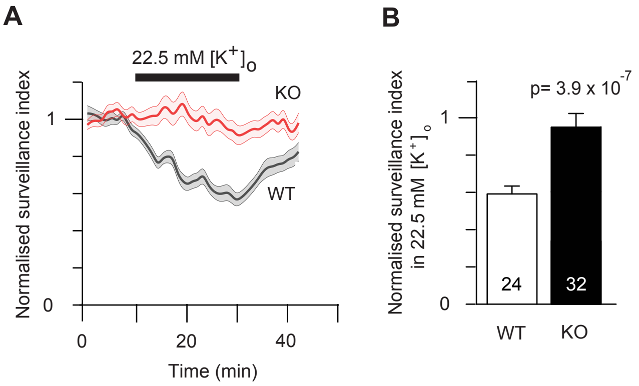

Supplementary Figure 3.

Depolarization by high [K+]o affects WT but not THIK-1 KO microglia (related to Figure 6).

(A) Time course of surveillance index (see STAR Methods), normalised to mean value in first 10 mins, for 24 microglia in brain slices from 3 P22-50 WT and 32 microglia in brain slices from 3 THIK-1 KO P22-31 mice during bath perfusion of solution raising $[K^+]_o$ from 2.5 to 22.5 mM (replacing Na^+; 0.5 µM TTX and 2 µM PSB-0739 were included in the solutions to block effects of changes in action potential frequency and ATP release evoked by bathing the whole slice in high $[K^+]_o$ solution). (B) Quantification of normalised surveillance index in last 5 mins of superfusion with high $[K^+]_o$.

Fig. S3

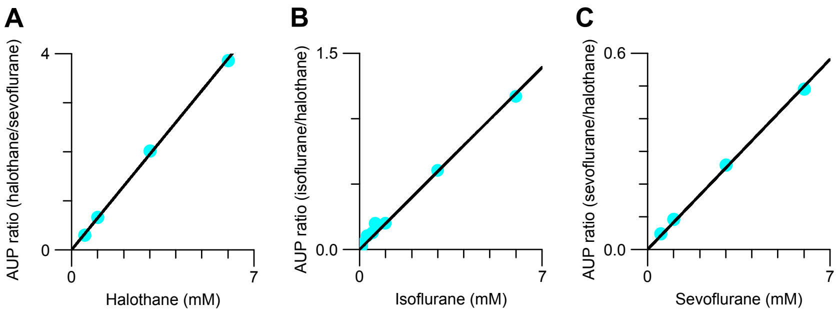

Supplementary Figure 4.
Quantification of anaesthetic concentrations (related to Figures 2 & 3).

GC-MS calibration curves were obtained by measuring the ratio of the area under the peak (AUP) detected for the tested anaesthetic, to the AUP of the 4 mM calibration standard (halothane or sevoflurane), with known concentrations of (A) halothane, (B) isoflurane and (C) sevoflurane. The R2 value for all three curves is > 0.99.

Fig. S4

A

Kcnk13-IN1-EM1-B6N sequence:

GCAGAGCAGGCTAAGGTCGGCAGAGCACATCCTCCCCGGGGCGTGGGCACAAGTCCGCGGGCAGCAACCCTC
GCGGAGCTGTCCTCCAGTGCCATGGCTGGCCGCGGTTGCGGCTGCAGCCCCGGCCACCTGAATCGAGGACAAC
GCGCGCTTCCTGCTGCTCGCTGGGCTCATCCTGCTCTACCTGCTGGGCGGCGCCGCAGTCTTCTCCGCGCTGGAG
CTAGCGCAGGAGTTGCAGGCCAAGCAGCGCTGGGAAGAGCGCCTGGCCAACTTCAGCCGGGGCCACAACCTG
AGCCGTGAAGAGCTGCGAGGTTTCCTCCGCCACTACGAGGAAGCCACCAGGGCGGGCATCCGCATGGACAGCG
TGCGCCCTCGCTGGGACTTCACGGGCGCCTTCTACTTCGTGGGTACAGTGGTTTCTACCATAGGTAAGTGAGCC
GCC

B

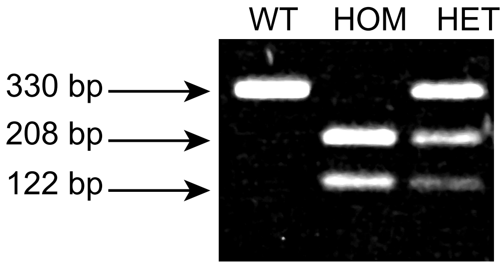

C

Kcnk13 wild type protein sequence:

MAGRGCGCSPGHLNEDNARFLLLAGLILLYLLGGAAVFSALELAQELQAKQRWEERLANFSRGHNLSREELRGFLRH
YEEATRAGIRMDSVRPRWDFTGAFYFVGTVVSTIGFGMTTPATTGGKIFLIFYGLIGCASTILFFNLFLERLITVIACVMR
SCHQQQLRRRGAVTQDNMKAPEKGEADSLTGWKPSVYYVMLILCLASVAISCGASALYTTMEGWSYFDSVYFCFVA
FSTIGFGDLVSSQNAQYESQGLYRFFNFFLILMGVCCIYSLFNVISILIKQTVNWILRKLDSGCFPPCQRGLLRSRRNVV
MPGNIRNRCNISIETDGVMESDTDGRRLSGEMISMKDTNKVSLAILQKQLSEMANGGPHQNSASSRDDEFSGGVG
AFAVMNNRLAETSGDR

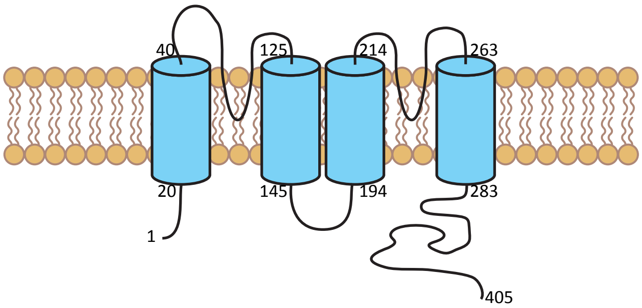

Kcnk13-IN1-EM1-B6N protein sequence:

MAGRGCGCSPGHLNRGQRALPAARWAHPALPAGRRRSLLRAGASAGVAGQAALGRAPGQLQPGPQPEP

1 68

Supplementary Figure 5.
Construction of THIK-1 KO mouse (related to Figures 2, 4, 5 & 7).

(A) Partial DNA sequence of KCNK13-IN1-EM1-B6N (THIK-1 knockout) showing the single nucleotide insertion in red. Genotyping primer binding regions are highlighted in blue. The frameshift caused by insertion of a thymidine residue results in the formation of a *TaqI* restriction site (yellow) which was used to identify the genotype following PCR amplification. (B) Agarose gel showing bands generated for wildtype (WT), THIK-1 homozygote knockout (HOM) and THIK-1 heterozygote (HET) mice, following PCR amplification using the primers described in A and restriction digestion with *TaqI*. (C) Peptide sequence and predicted protein structure for THIK-1 (KCNK13 wild type, shown in lipid membrane) and for the THIK-1 knockout (lacking a transmembrane domain, bottom line). Transmembrane domains are highlighted in green. Amino acids in red are those generated due to the frameshift caused by the insertion. Hydropathy analysis indicates that this sequence is of low complexity and will not form a transmembrane domain. Numbers represent amino acid positions.

Fig. S5
